# Role of Slow-Release Nanocomposite Fertilizers on Nitrogen and Phosphate Availability in Soil

**DOI:** 10.1038/srep46032

**Published:** 2017-04-13

**Authors:** Amanda S. Giroto, Gelton G. F. Guimarães, Milene Foschini, Caue Ribeiro

**Affiliations:** 1 Federal University of São Carlos, Department of Chemistry, Washington Luiz Road km 235, Zip Code: 13565-905 São Carlos, SP, Brazil; 2 Embrapa Instrumentation, 1452, XV de Novembro Street, CP: 741, Zip Code: 13560-970 São Carlos, SP, Brazil

## Abstract

Developing efficient crop fertilization practices has become more and more important due to the ever-increasing global demand for food production. One approach to improving the efficiency of phosphate and urea fertilization is to improve their interaction through nanocomposites that are able to control the release of urea and P in the soil. Nanocomposites were produced from urea (Ur) or extruded thermoplastic starch/urea (TPSUr) blends as a matrix in which hydroxyapatite particles (Hap) were dispersed at ratios 50% and 20% Hap. Release tests and two incubation experiments were conducted in order to evaluate the role played by nanocomposites in controlling the availability of nitrogen and phosphate in the soil. Tests revealed an interaction between the fertilizer components and the morphological changes in the nanocomposites. TPSUr nanocomposites provided a controlled release of urea and increased the release of phosphorus from Hap in citric acid solution. The TPSUr nanocomposites also had lower NH_3_ volatilization compared to a control. The interaction resulting from dispersion of Hap within a urea matrix reduced the phosphorus adsorption and provided higher sustained P availability after 4 weeks of incubation in the soil.

Nitrogen (N) and Phosphorus (P) are two important macronutrients responsible for the growth and yield of agricultural crops. Developing efficient fertilization practices has become more and more important due to the ever-increasing global demand for food products. About 40–70% of N and 80–90% of P applied as normal fertilizers are lost to the environment or chemically bound in the soil and are unavailable to plants^1^,^2^,^3^,^4^,^5^. These losses come at a large economic and environmental cost. The low efficiency of P fertilizers is attributed to the formation of Fe- and Al-based oxides, especially in tropical soils. Most of the P released from organic matter and that added as fertilizer is rapidly scavenged by soil minerals and turned into fixed or insoluble inorganic compounds that are not susceptible to leaching^6^. Thus, soil PO_4_^3−^ concentrations are typically very low (less than 0.01 to 1.00 ppm)^7^,^8^. On the other hand, high NH_3_ volatilization due to a rapid hydrolysis of urea, leads to an accumulation of NH_4_^+^ and increase in soil pH.

Recently, the use of slow release fertilizers (SRF) has been considered to be a promising strategy to improve the utilization of macronutrients^4^,^9^,^10^. Slow-release fertilizers have many advantages over conventional fertilizers, confirmed in different cultures such as improved fertilizer use efficiency in potato^11^,^12^, better matching of nutrient demand in crops^13^, and increased P recovery in barley^14^. Many studies propose novel developments, such as attapulgite-based fertilizers^15^,^16^ or urea-templated oxalate-phosphate-amine a novel slow-release fertilizer^6^. The overall effect is that less fertilizer is needed which lowers the potential negative effects from over-fertilization^17^,^18^. Beyond this, slow release fertilizers are designed to release their nutrient contents gradually in order to coincide with the nutrient requirement of plants. These fertilizers can be physically prepared using engineered matrices as a way to control their release rate^9^,^19^. Currently, degradability of attention towards environmental protection issues^20^. Furthermore, matrices must be compatible with the surface properties of fertilizers, i.e., they should be hydrophilic. As previously proposed by our group^19^, interesting candidate matrices for SRF are thermoplastic starch and urea. Urea is one of the most important synthetic fertilizers worldwide due to its low cost and high N content. On the other hand, starch is a cheap, largely available and biodegradable natural polymer that has been extensively used as an encapsulating matrix of agrochemicals. When plasticized by alcohols or even by urea, starch is known as thermoplastic starch (TPS). Composite materials made of starch and urea have not been successfully tested as a conventional urea-based fertilizer displaying a prolonged N release profile.

In addition to controlling the release of urea, the starch matrix could improve the efficiency of phosphate fertilization. The combination of urea and phosphate within a single matrix has the potential of decreasing P fixation in the soil^6^. The high pH created in the starch matrix from the conversion of urea to NH_4_^+^ may able to change the dynamic of chemical fixation of phosphorus in the soil^21^. Therefore, the dispersion of phosphate-rich minerals into urea and thermoplastic starch as a nanocomposite produced by extrusion could provide control over the release of urea and increase the availability of P in soil.

In the present work, we report on a strategy to use nanocomposites produced from TPS, urea, and phosphate as a slow release fertilizer matrix. The synergistic roles played by the host matrices in preventing immobilization of phosphate and volatilization of nitrogen when in contact with soil were studied in detail.

## Results and Discussion

Table 1 shows the content of P and N in the pure urea, single superphosphate (SSP), hydroxyapatite (Hap) and in the nanocomposites. The nanocomposites presented reduced of nutrient contents in relation to the pristine materials due to incorporation of Hap in urea or TPSUr polymer. Their N and P contents were verified to fall between 3 and 8% and from 8.4 to 38%, respectively, which are amounts comparable to those comprised in commercial fertilizers.

**Figure 1:**
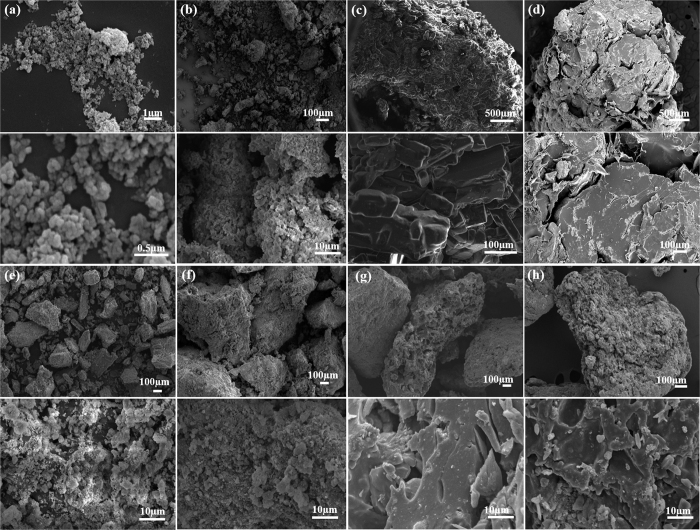
SEM images of the materials precursors (**a**) Hap, (**b**) SSP, (**c**) Urea, (**d**) TPSUr, (**e**) UrHap20, (**f**) UrHap50, (**g**) TPSUr/Hap20 and (**h**) TPSUr/Hap50.

Figure 1 shows SEM images of the nanocomposites, as well as the morphologies of pure Hap and SSP (materials used in control experiments). As observed in Fig. 1(a), Hap powder is comprised of nanometric particles with agglomerate sizes around 100–300 nm. SSP powder is characterized by micrometric agglomerates, Fig. 1(b). However, careful observation of Fig. 1(b) reveals the nanometric structure of SSP which is characterized by many small particles in the agglomerate. These features may influence the solubilizing profile of both materials since the dissolution dynamics is dependent on the surface accessibility – which is related to particle size. In Fig. 1(c), the characteristic morphology of urea appeared crystallized as platelets with sizes larger than 100 μm. The SEM micrograph of TPSUr in Fig. 1(d) exhibits a relatively homogeneous surface, indicating the efficiency of urea in plasticizing starch.

All nanocomposites showed a complex structure, which is clearly seen in Fig. 1(e,f,g and h). TPSUr and urea matrices produce a continuous phase in which Hap nanoparticles are embedded, and generally, phase separation is not observed. In this case, the nanocomposite samples can be assumed to be homogeneous, although TPSUr is characterized by a continuous, porous structure, whereas urea produces a more granular, friable matrix – as seen by the small particle sizes obtained.

**Figure 2:**
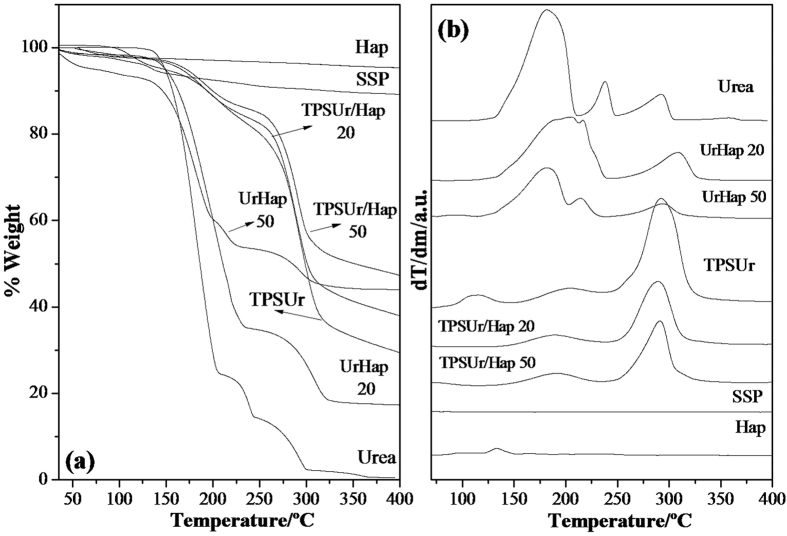
(**a**) Thermogravimetric analysis of materials and (**b**) derivate.

Figure 2 shows the thermogravimetric profiles of the pristine materials and nanocomposites. It is possible to see that Hap presents a weight loss of only 5% while SSP has a maximum weight loss of 10%^22^. These thermal events are related to elimination of physically bonded and structural water molecules (Fig. 2a). TPSUr shows the typical degradation curve of urea-plasticized starch with appearance of a final residue content of 30%, possibly due to strong interaction between urea and starch^23^,^24^. Four of the most significant decomposition stages of pure urea were identified by Chen and Isa^25^. The first weight loss starts at 118 °C before the urea melting point (133 °C) until complete urea oxidation at around 380 °C. The nanocomposite UrHap50 shows a thermal degradation behavior largely represented by the urea degradation, thus showing the same degradation temperatures. The nanocomposite UrHap20 comprised the lowest Hap content but presented the highest dispersion of urea within the matrix. This proves that high Hap dispersion accounted for the increased thermal stability of the nanocomposites, as seen from the changes in the temperature of the first urea degradation stage, at about 20 °C, in Fig. 2(b). This behavior is due to strong interactions between Hap nanoparticles and urea, as signs of degradation related to free urea are not observed in the thermal profiles of the nanocomposites. TPSUr/Hap50 and 20 have a thermal degradation behavior mainly represented by degradation of the TPSUr polymer matrix. A decrease of about 8 °C was observed in the thermal stability of starch phase of TPSUr/Hap20 when compared with the degradation peak of the pure polymer network (Fig. 2(b)). This is probably because of small polar molecules inside the structure that would accelerate the breakdown of starch chains, as reported by Giroto *et al*.^26^. The final residue in all nanocomposites refers to the Hap phase when present.

**Figure 3:**
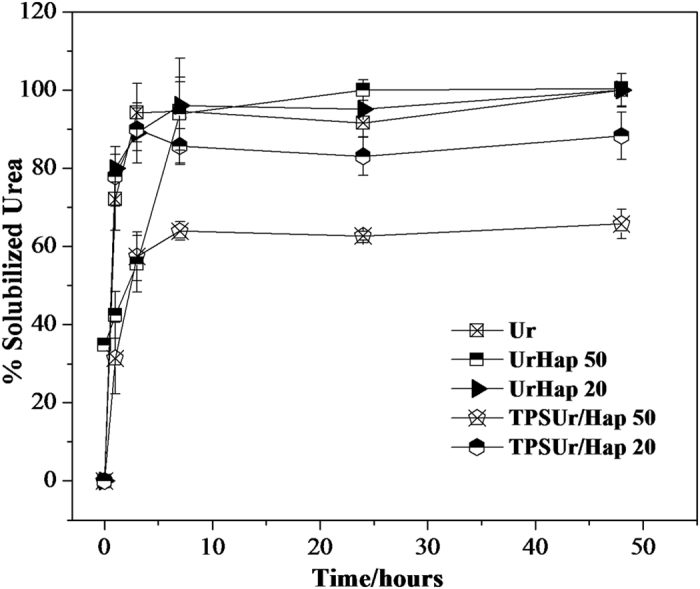
The release rate of urea in water as a function of time for pure urea and each of the composites at pH 7 and 25 °C.

The use of urea as dispersing matrix for Hap was an interesting choice since urea is highly water soluble and is of great agronomic importance. Release rates (produced in Fig. 3) were then determined to assess the functionality of the nanocomposites on urea solubilization. It can be observed rapid dissolution of pure urea within only two hours. The nanocomposites UrHap50 and UrHap20 displayed solubilizing behavior very similar to that of pure urea, and reached equilibrium between 10–15 h. A slower urea release behavior was observed for the nanocomposites based on TPSUr matrix. The nanocomposite containing 50% Hap (TPSUr/Hap50) provides a solubilizing urea extent of only 55%. Additionally, it is possible to verify a different behavior for the nanocomposite TPSUr/Hap20. The presence of Hap induced the formation of pores throughout the nanocomposite granule structure. These pores allowed the solution to flow out, which facilitated solubilizing of urea comprised in the polymer matrix. It can be seen that part of urea used to plasticize starch ended up being encapsulated by the TPSUr matrix, thereby reducing its contact with water.

**Figure 4:**
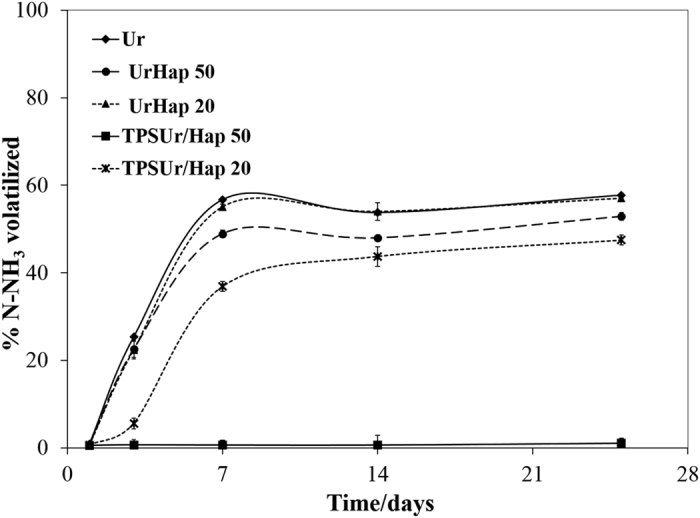
Percentage of volatilized ammonia (NH_3_) during the aerobic incubation of urea or composites applied to the soil. Data are shown as a mean of triplicate incubations with bars to indicate the standard deviation of the mean.

Figure 4 shows ammonia volatilization results from urea or nanocomposites soil incubation experiments. The rapid N-release from urea or nanocomposites UrHap50 and UrHap20 in soil can be observed by the intense NH_3_ volatilization that occurred over 7 days of incubation, with values of N loss equal or higher than 50% in relation to the total N applied as urea. In contrast, NH_3_ volatilization losses for nanocomposites TPSUr/Hap20 did not exceed 46% until the second week of incubation, indicating a slower N-release behavior. This finding can be attributed to the controlled release of urea by the thermoplastic starch (TPS), the amended urea TPSUr/Hap50 presented the higher controlled behavior with inexpressive NH_3_ volatilization during the incubation period. As evidenced in Fig. 3, the urea encapsulated by the TPS matrix had reduced contact with the solution. Moreover, the highest rate of TPSUr/Hap50 compared to TPSUr/Hap20 processed by extrusion promoted a greater control over the NH_3_ volatilization by the lower N-release extent or by some interaction between ammonium and phosphates in soil. A little advantage was evidenced for the nanocomposite UrHap50 with respect to N losses in relation to the unamended urea, which may be attributed to the same interaction. The high NH_3_ volatilization rate (about 50% of the N-urea applied) in this soil could be attributed to the rise of pH caused by urea hydrolysis, combined with the low soil CEC and buffer capacity, which created conditions favorable for NH_3_ volatilization. This behavior was also observed for Guimarães *et al*.^27^. The authors worked with similar chemical-physical characteristics to the soil used in this work and using the similar incubation system. They observed a N losses from unamended urea ultimately exceeded 50% for the Assis soil (CEC of 3.5 cmolc kg^−1^ and pH 5.3), as compared to more than 70% for the Bloomfield soil that was lower in CEC (1.2 cmolc kg^−1^) and higher in pH 6.9. In addition, the incubation system utilized can be intensified the NH_3_ volatilization due to the limited diffusion of N in the small sample of soil used this incubation experiments.

The volatilization model of ammonia was well adjusted to all nanocomposites, except for TPSUr/Hap50 which showed inexpressive N losses over the incubation intervals (Table 2). Following a Logistic model – *y* = *A*. (*1* + *B.e*^−*C.t*^)^−*1*^, where A is the maximum NH_3_ volatilization expected (or total N volatilized); C is a recovery factor and B is the weight of delayed release on the total volatilization. In this model, when C > 1, the release tends to the conventional release, whereas when 0 < C < 1 the delays are more representative on the total profile.

The unamended urea sample exhibited the highest loss of total N as NH_3_ volatilization and it differed from the other composites, UrHap50 and TPSUr/Hap20. However, it is similar to that of nanocomposite UrHap20. The nanocomposite TPSUr/Hap20 showed a reduction of 10% of the N loss compared with urea. Moreover, the NH_3_ losses occurred more gradually from TPSUr/Hap20, requiring 5.5 days to volatilize 25% of the total N added to soil, whereas unamended urea required 2.9 days to reach the same volatilization extent. Further evidence of controlled volatilization is the lower C recovery factor for the nanocomposite TPSUr/Hap20 in relation to the other treatments. The latter finding shows that TPS was effective in controlling the urea release, however, this process was influenced by the rate of Hap, as evidenced in the nanocomposite TPSUr/Hap50, which showed only 1% of the N volatilized due to the very low urea release extent. The advantage of the UrHap50 nanocomposite, cannot be attributed to the control urea release, since both treatments volatilized 25% of the total N incubated in the soil in same time.

**Figure 5:**
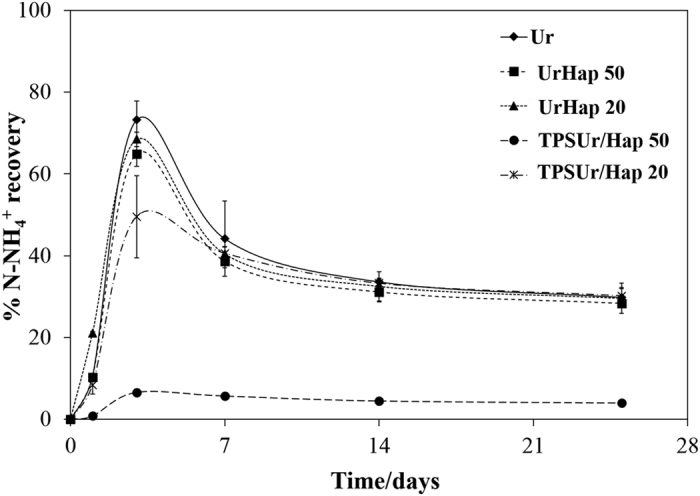
N recovery as ammonium (NH_4_^+^) during the aerobic incubation of urea or nanocomposites applied to the soil. Data shows as a mean of triplicate incubations with bars to indicate the standard error of the mean.

Figure 5 compares the five treatments in terms of N recovered as exchangeable NH_4_^+^ before and after incubation intervals. N recoveries values reported in Fig. 5 indicate production of NH_4_^+^ through urea hydrolysis after release of unamended urea or nanocomposites, and consumption by NH_3_ volatilization. The NH_4_^+^ levels had maximum accumulation until the third day of incubation. However, the increase in NH_3_ volatilization after 3 to 7 days (Fig. 4) led to a gradual decrease in NH_4_^+^ recovery continued throughout the incubation period. When compared to pure urea, the nanocomposite TPSUr/Hap20 exhibited significantly lower exchangeable NH_4_^+^ content after 1 and 3 days of incubation, but this difference became insignificant after the first week of incubation. The lower NH_4_^+^ content of the nanocomposite TPSUr/Hap50 may be attributed to its very low urea release. It is noteworthy that the recovered N as NH_4_^+^ for samples Ur, UrHap50 and UrHap20, are consistent with the final NH_3_ volatilization: in general, the unrecovered N corresponds approximately to the volatilized content. On the other hand, the sample TPSUr/Hap20 presented a final N recovery of 30% and a volatilized NH_3_ content of 45%. This indicates that a significant N amount still remains into the nanocomposite structure, and could be released over longer times. In fact, this result is consistent with the lower recovery factor of this sample (C = 0.87), as shown in Table 2. Finally, the very low N recovery values for the nanocomposite TPSUr/Hap50, associated with the negligible NH_3_ volatilization, strongly suggests that this sample is still undergoing the N releasing process.

Owing to ammonium content in soil is a result of urea hydrolysis and ammonia volatilization process, kinetics parameters of NH_4_^+^ recovery were estimated to study the N transformation within the soil (Table 3). The samples Ur, UrHap20 and UrHap50 presented similar maximum recovery of NH_4_^+^, which exceeded 75% of the total N incubated in the soil, and occurred at the same time, between 2.2 and 2.9 days. However, the nanocomposite TPSUr/Hap20 showed a lower maximum NH_4_^+^ recovery and delayed the formation of ammonium in the soil in relation to unamended urea. The nanocomposite TPSUr/Hap50 showed the lowest formation of NH_4_^+^, indicating that TPS and Hap prevent the urea release. The values below the tangent curve at 6 days indicate that the slowed transformation of ammonium to ammonia in the soil and the NH_4_^+^ content was more homogeneous over the incubation period. The ammonium contents after the incubation period (25 days) were similar, between 28.3 to 30.2% of the N applied to soil, except for the nanocomposite TPSUr/Hap50, which had a low urea release profile. The latter finding may be attributed to the little soil ability to retain ammonium as its CEC and buffer capacity are low, which standardized the ammonium content in the soil. Although the nanocomposite TPSUr/Hap20 presented ammonium content similar after the incubation period, the control over the urea release provided a smaller N loss by volatilization and maintained a more constant NH_4_^+^ content in the soil.

The rational model can be interpreted as a balance of a solubilizing curve (from fertilizer), which is the only source of NH_4_^+^, and volatilization – as shown in Fig. 4. In fact, the content at equilibrium is a soil characteristic, and may vary over time. Then, during 3–7 days, all the samples presented a metastable feature, i.e., this NH_4_^+^ remained available to the soil but started to volatilize because it was not used in any fixation process.

Table 4 shows the recovery of NO_3_^−^ during aerobic incubation of urea or nanocomposites in soil. The low nitrate level during incubation can be attributed to limited nitrification of NH_4_^±^, which did not exceed 2% from N-urea incubation soil and did not present a consistent profile in evaluated period. No definite explanation can be offered for the latter finding. However, the low nitrification in coarse-textured soils was also reported by Guimarães *et al*.^27^, using the similar incubation system. The authors report that nitrification was limited by the extensive loss of urea-derived NH_4_^±^-N through volatilization. The high concentration of ammonia (NH_3_) inhibits the activity of bacteria of the genus *Nitrobacter* responsible for nitrification from NO_2_^−^ to NO_3_^−^ 28.

**Figure 6:**
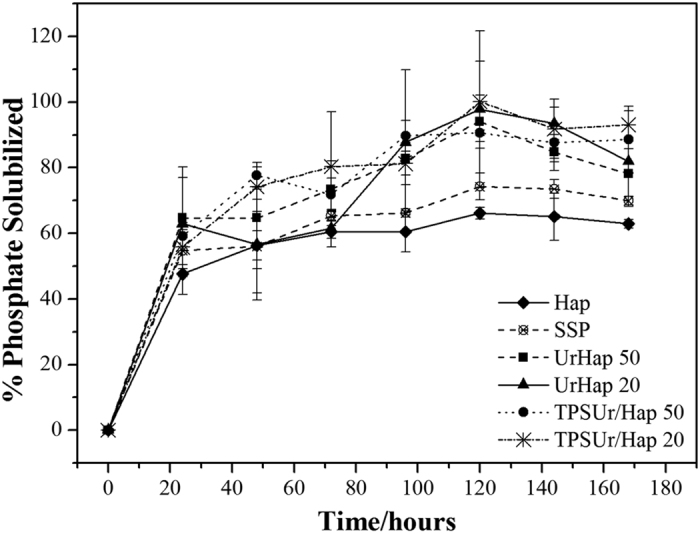
The release rate of phosphorus in citric acid solution (2 wt.%) as a function of time for Hap, SSP and each of the nanocomposites at pH 4 and 25 °C.

The solubilizing curves of Hap for the nanocomposites were compared with those of Hap and SSP, as represented in Fig. 6. Analyzing the behavior of the nanocomposites, it can be noticed that all samples presented a higher Hap release compared to pure Hap and SSP. This means was increase of over 40% for all composite total release time for Hap and SSP, which both released at the same time only 60 and 68% Hap respectively.

It can be proved that the morphology of all nanocomposites before grinding was effectively responsible for the phosphate solubilization. The nanocomposite matrix containing urea had a dense structure in which the solubilization of Hap occurred only after complete solubilization of the urea structure. On the other hand, the TPSUr/Hap nanocomposites presented faster releases of phosphate and urea than the Ur/Hap nanocomposites, which is probably due to the water accessibility through their highly porous matrices. In fact, this is possible because the porous structure of the TPSUr/Hap nanocomposites could facilitate the release of phosphate at short times, but the structure is likely to be modified by swelling, obstructing the water transport over longer times^19^. The original structures of the materials were modified by grinding and therefore their release profiles were modified as well. As can be seen from Fig. 6, there is no significant difference between the total amounts of Hap released for each nanocomposite.

The P-source release from Hap, SSP or nanocomposites applied to soil was evaluated after 42 days of aerobic incubation with controlled temperature and humidity. The fraction of available phosphorus (labile) in the soil after the incubation period and at the start of incubation (0 day) was extracted in water and anionic resin. The availability of P in the soil showed a similar behavior for both extractors, but with different extents. The anion exchange resin promoted a large extraction of the available P fraction, with values between 44 and 97% of the total P applied to the soil, whereas water extracted a small part of the available P fraction, with values between 1 and 26%.

Table 5 shows the fraction of available P in the water at the start (0 day) and after incubation period (42 days), relative to the total P incubated in soil. The SSP and Hap had similar water solubility at the start of incubation, an average of 26 and 21% of the total P, respectively. The dispersion of Hap within the urea matrix, as in the nanocomposites UrHap50 and UrHap20, did not increase the release of P compared with pure Hap. Conversely, the TPSUr matrix decreased the release of P from Hap. This result could be attributed to the high water solubility of Hap, similar to that of SSP, which did not reflect to the effect of dispersion of the soluble matrix as urea, when applied to the soil. The TPSUr array can be assigned to the retention effect and control of release provided by the polymer. It acted as a physical barrier, retarding release of P to soil, different from that observed in the release solution of 2% citric acid (Fig. 6).

After the incubation period (42 days), there was a reduction of the labile fraction of P in soil extracted with water for SSP and Hap, in comparison with the P available at the start of the incubation. This reduction can be attributed to the P adsorption by soil colloids. The Oxisol used in the incubation experiment has favourable characteristics that are indicative of high adsorption of P in soil, such as low pH, low available P content, low P remaining and high acidity potential^29^. Additionally, the prevalence of Fe and Al oxides in Oxisol makes them more effective with regard to adsorption of phosphoros^30^.

Nanocomposites UrHap50 and UrHap20 showed greater P availability after the incubation period, showing a decreased adsorption of P by the soil compared with SSP and Hap. This result can be explained by two factors: the first is related to raise of pH of the soil around the particles due to urea hydrolysis, which can reach pH between of 8 to 9^31^,^32^,^33^. The second factor is the interaction between the NH_4_^+^ ions formed during the hydrolysis of urea and anion P release from Hap, providing a reduction in the adsorption of phosporus^21^.

Table 6 shows the pH change after the incorporation of the fertilizers or nanocomposites in the soil at the initial time (0 day) and 42 days after the incubation. The soil pH increased for Hap and composites treatments at the initial time, that can be attributed to the higher pH of these sources (pH ≈ 6 for Hap and 7 for urea) compared to the initial soil pH (pH = 5.19). On the other hand, it was observed a small decrease in the soil pH after SSP incorporation, related to its acid character. After 42 days of incubation, the pH of the soil fertilized with Hap or SSP remained close to the pH of the soil without fertilization, with pH around 5. However, in this same period, the soil fertilized with the nanocomposites preserve higher pH in relative of the soil without fertilization, with values between 6.30 to 7.90. The latter findings are to be expected due the high hydrolyses of urea in the soil with low CEC and buffer capacity.

Thus, the results indicate that the interaction between urea and Hap, both nanocomposites UrHap50 and UrHap20, provided a more homogeneous phosphorus availability during the incubation period. On the other hand, nanocomposites TPSUr/Hap that have lower urea content and control of P release had lower availability of P and greater heterogeneity.

As previously mentioned, the available P estimated by anionic resin showed the same behavior of P extracted by water of different treatments, however, the resin provided the extraction of a larger fraction of P-labile (Table 5). After 42 days of incubation of Hap and SSP in soil, the P availability decreased by 42 and 52%, respectively, demonstrating the high adsorption P in the soil under these conditions, as discussed above. On the other hand, nanocomposites containing Ur/Hap showed little variation during the same period and presented a high availability of P with values between 82 and 97% relative to the total P applied to the soil. Similarly, the nanocomposites containing TPSUr/Hap also had the most homogeneous phosphorus availability during the incubation period, but with lower values ranging between 62 to 76%.

## Methods

### Raw materials

The raw materials used in the nanocomposite formulations were: urea (Synth, Brazil), hydroxyapatite (Sigma-Aldrich, USA), Amidex 3001 (Corn Products, Brazil) citric acid (Synth, Brazil), and stearic acid (Vetec, Brazil). A commercial simple superphosphate (SSP) (Heringer, Brazil) was used for comparison purposes. Other materials were used as received.

### Preparation of Nanocomposites

The nanocomposites having urea as a matrix were prepared by mixing urea (Ur) and hydroxyapatite (Hap) at 20 and 50 wt.% Hap ratios (w.w^−1^ basis) using a urea mixed-melt processing method. The nanocomposites were produced on a torque rheometer (Polylab RHEODRIVE Rheomix mixer and OS4) under conditions of 60 rpm for 10 min at 100 °C, and further dried at room temperature for 24 hours. These nanocomposites were designated as UrHap50 and UrHap20. The nanocomposites comprising thermoplastic starch (TPS) as a matrix were obtained from a physical mixture of corn starch, urea (Ur) and distilled water at a mass proportion of 56/24/20, respectively. Stearic acid 1% (w.w^−1^) and 1% citric acid (w.w^−1^) were added to this blend. This final formulation was processed on a co-rotating twin screw extruder (L/D = 40, ZSK-18 Coperion model) equipped with conveying and kneading elements. Six heating zones on the extruder were set at temperatures of 100, 110, 115, 120 and 120 °C. The extruder was operated with a rotating speed of 150 rpm and the extrudate was extruded through a rod die to obtain TPSUr (TPS + Ur) blends in the form of rods that were subsequently pelletized. The TPSUr/Hap nanocomposites with Hap mass contents of 50 and 20 wt% were produced by mixing and processing the powders (starch, urea, stearic acid, citric acid, and Hap) by extrusion using the same processing conditions used to obtain the pure TPSUr blend. These nanocomposites were designated as TPSUr/Hap50 and TPSUr/Hap20, respectively. Both types of materials have been previously described by Giroto *et al*.^19^.

### Characterizations

Scanning electron microscopy (SEM) was performed on a JSM6510 microscope (JEOL) (Thermo Scientific NSS coupled or linked). Samples were previously fixed onto carbon stubs and coated with thin layer of gold in an ionization chamber (BALTEC Med. 020). SEM imaging was carried out using the secondary electron mode. Thermal degradation of samples was evaluated in the range 25 °C–600 °C using a Q500 analyzer (TA Instruments, New Castle, DE, USA) under the following conditions: sample size of 10.0 ± 0.5 mg, synthetic air atmosphere (80% N_2_ and 20% O_2_) with flow of 60 mL.min^−1^, and heating rate of 10 °C.min^−1^.

### Release Tests in solution

Determination of phosphorus content was based on the method reported by Murphy and Riley^34^. An apparatus was arranged by placing different mass of each sample into a beaker with capacity of 600 mL containing citric acid solution 2% (w.w^−1^) in order to obtain the same final concentration (250 mg L^−1^) of phosphorus for each material. The mixture was incubated and kept in a chamber at controlled temperature of 25 °C and agitation of 45 rpm (347 Fanem CD). Aliquot parts were collected at different time intervals up to 144 hours for phosphate quantification in triplicate. Concomitantly, quantifications were performed for tests involving pure Hap and SSP as a control experiment. Urea release experiments were performed parallel with phosphorus solubilization tests according to the method adapted from Tomaszewwska and Jarosiewicz^35^. The concentration of urea in solution was determined by UV-Vis spectrophotometer (Shimadzu-1601PC). Each measurement was done in triplicate under identical experimental conditions for each sample. The concentration final of urea (1250 mgL^−1^) also was standardized in order to have the same final concentration of urea for all materials.

### Release and transformation of Nitrogen in Soil

Release of urea from nanocomposites was evaluated in a Red-Yellow Oxisol which was collected at 20 cm depth at a pasture site in São Paulo. The soil was previously dried in air and sieved through a 2 mm screen. Physical-chemical characterizations provided the following soil parameters: 667 g kg^−1^ (sand), 19 g kg^−1^ (silt) and 314 g kg^−1^ (clay), according to soil texture analysis by the pipette method^36^; water-holding capacity^37^ of 140 g kg^−1^; pH (H_2_O) 5.3, measured with a glass electrode; organic C content of 7.5 g kg^−1^ by the Walkley-Black method^38^; available P content of 1.9 mg kg^−1^, total N content of 1.06 g kg^−1^ by Kjeldahl method^39^; cation-exchange capacity (CEC)^36^ of 4.77 cmolc kg^−1^, remaining phosphorus^40^ (P-rem) content of 20 mg L^−1^, potential acidity^15^ (H + Al) of 3.8 cmolc kg^−1^ and urease activity of 9.63 mg urea N hydrolyzed kg^−1^ soil h^−1^ by a modified Tabatabai and Bremner buffer method^41^.

The incubation system used was similar in design to the unit described by Bremner and Douglas^42^ with the modifications proposed by Guimarães *et al*.^27^. Samples of soil (10 g^−1^ sample) were incubated with urea or nanocomposite at a soil:N ratio of 1000:1 (g g^−1^) in polyethylene bottles com capacity of 125 mL. The samples were weight with this amounts (Ur: 21.32 mg g^−1^; UrHap20 26.60 mg g^−1^; UrHap50 41.70 mg g^−1^; TPSUr/Hap20 87.34 mg g^−1^ and TPSUr/Hap50 118.80 mg g^−1^). Samples were incorporated into the soil which was humidified to 80% of its water retention capacity with the addition of deionized water. A container with 5 mL of 4% boric acid solution was added to the polyethylene bottles for scavenging the volatilized ammonia (NH_3_) during the incubation period. Incubation was performed for 0, 1, 3, 7, 14, 25 days under controlled temperature and relative humidity.

Volatilized NH_3_ was quantified by titration of boric acid with HCl (0.01 mol L^−1^). Mineral N produced during incubation was extracted by shaking the soil sample with 100 mL of KCl (l mol L^−1^) containing phenyl mercuric acetate (5 mg L^−1^) as a urease inhibitor (soil:solution ratio of 1:10). Afterwards, the suspension was kept under stirring for 1 hour and filtered with slow filter paper (diameter 12.5 cm). The resulting soil extract was stored in 100 mL polyethylene bottles at 5 °C.

The ammonium (NH_4_^+^) and nitrate (NO_3_^−^) levels in the soil extracts were determined by the colorimetric methods of Kempers and Zweers^43^ and Yang *et al*.^44^, respectively. The contents recovered in each N fraction were expressed as percentages in relation to the N added to soil in the form of urea or nanocomposite. Replicate data of NH_3_ were adjusted to the *Logistic model*, which allowed estimating the total content of volatilized N and the time required to volatilize 25% of the N added to the soil. Recovery of N as NH_4_^+^ in soil was adjusted to the *Rational model*, which permitted estimating the maximum recovery of NH_4_^+^, time of maximum NH_4_^+^ recovery, curve tangent at 6 days and the NH_4_^+^ recovery at the end of the incubation. The adjusted models were presented by their average values and respective standard deviations. Differences between means values were determined by analysis of variance (ANOVA). Duncan´s multiple comparison test was used to compare all pairs of treatments previously determined as significant by the F test. The significance level was set at 0.05.

### Release and Availability of Phosphorus in Soil

The release of phosphorus to soil was evaluated using the Red-Yellow Oxisol described in the previous section. Soil samples (50 g sample^−1^) were incubated with Hap, SSP or nanocomposites at soil:P ratio of 5000:1 (g g^−1^) in 300 mL capacity transparent plastic bags. Distilled water was then added to raise the soil moisture to 80% of its moisture holding capacity. Samples were incubated for 42 days at controlled temperature and relative humidity. Soil moisture was monitored throughout all experiment and distilled water was added to maintain the moisture at 80% when necessary.

After the incubation period, the soil samples were dried in air and sieved through a 2 mm screen. The available P was extracted with water and anionic resin as proposed by Quaggio and Raij^45^. The extraction of P with water was performed at water:soil rate of 1:5). 5 g of soil were stirred in 25 mL of water for 30 min in 50 mL capacity using Falcon tubes. After stirring, the suspension was centrifuged at 3000 rpm for 15 min and the supernatant (extract) was collected for available P determinations. This extraction procedure was repeated three times. The contents of P (P-water) and (P-resin) were measured by molecular absorption spectrometry. The available P content of the soil at the initial time of incubation (0 day) was also determined. The pH (H_2_O) of soil was measured (soil:water ratio, 1:2, 5) with a glass electrode of samples at inicial time (0 day) and 42 days after incubation.

Available P contents were expressed as a percentage of the total P applied to the soil. The percentages of P extracted with water were expressed as a sum of three successive extractions. Differences between means values were determined by analysis of variance (ANOVA) and F test. Differences between pairs of treatment means were determined by Duncan-test at significance level of 0.05.

## Conclusion

The control over the nutrient release and homogenous availability of nutrients in soil are desirable characteristics of fertilizers that have promoted increasing interest in research. The strategy involving dispersion of mineral phosphate sources into urea and thermoplastic starch (TPSUr) have great potential, allowing the control N-release and increasing P-availability in soil. In the conditions tested in this work, the interaction between Hap and urea are probably the responsible factors that reduced phosphorus immobilization and consequently provided greater P availability in soil during the incubation period.

## Additional Information

**How to cite this article:** Giroto, A. S. *et al*. Role of Slow-Release Nanocomposite Fertilizers on Nitrogen and Phosphate Availability in Soil. *Sci. Rep.* **7**, 46032; doi: 10.1038/srep46032 (2017).

**Publisher's note:** Springer Nature remains neutral with regard to jurisdictional claims in published maps and institutional affiliations.

## Figures and Tables

**Table 1:** The content of P and N in the nanocomposites.

Composites Fertilizers	P (g Kg^−1^)	N (g Kg^−1^)
Urea	—	450
SSP	100	—
Hap	150	—
UrHap50	80	240
UrHap20	40	380
TPSUr/Hap50	50	84
TPSUr/Hap20	30	115

**Table 2:** Model adjusted for volatilization of ammonia (NH_3_), total nitrogen volatilized in the incubation period, and time spent to volatilize 25% (Time 25%) of the N applied to the soil from urea or composites.

Treatment	Logistic Model	Total N volatilized	Time 25%
		%	days
Ur	ŷ = 56.1/(1 + 640.4 e ^(−2.08 t)^)	56.1 d^1^	2.9 a
UrHap50	ŷ = 50.0/(1 + 513.2 e ^(−2.01 t)^)	50.0 c	3.1 a
UrHap20	ŷ = 55.4/(1 + 840.6 e^(−2.03 t)^)	55.4 d	3.1a
TPSUr/Hap50	—	1.0 a	—
TPSUr/Hap20	ŷ = 45.6/(1 + 133.3 e ^(−0.87 t)^)	45.6 b	5.5 b

^1^Mean values reported from triplicate incubations. Values within a column followed by the same letter do not differ significantly by the Duncan’s test at a significance of 0.05.

**Table 3:** Model adjusted for N recovery as ammonium, maximum recovery NH_4_^+^ (max NH_4_^+^), time that occurred the max NH_4_^+^ (t max), tangent curve in 6 days (tg 6), and recovery NH_4_^+^ in the final incubation period (Final NH_4_^+^) of urea or nanocomposites in soil.

Treatment	Rational models	max NH_4_^+^	t max	tg 6	Final NH_4_^+^
		%	days		%
Ur	ŷ = (0.5t^2^)/(1−0.71t + 0.19t^2^)	77 c^1^	2.9ab	−0.46 c	29.8 b
UrHap50	ŷ = (0.81t^2^)/(1−0.92t + 0.32t^2^)	76 c	2.2 a	−0.31 b	28.3 b
UrHap20	ŷ = (0.83t^2^)/(1−0.92t + 0.32t^2^)	79 c	2.2 a	−0.33 b	29.6 b
TPSUr/Hap50	ŷ = (0.05t^2^)/(1−0.54t + 0.14t^2^)	07 a	3.8 c	−0.04 a	4.0 a
TPSUr/Hap20	ŷ = (0.5t^2^)/(1−0.59t + 0.19t^2^)	52 b	3.5bc	−0.25 b	30.2 b

^1^Mean values reported from triplicate incubations. Values within a column followed by the same letter do not differ significantly by the Duncan’s test at a significance level of 0.05.

**Table 4:** Recovery of N as exchangeable nitrate (N-NO_3_^−^) during aerobic incubation of urea or nanocomposites in soil.

Treatment	Recovery of NO_3_^−^ after incubation period (days)/%				
	1	3	7	14	25
Ur	0,64 ± (0,05)^1^	0,83 ± (0,10)	1,57 ± (0,03)	1,56 ± (0,05)	0,80 ± (0,03)
UrHap50	0,78 ± (0,02)	1,77 ± (0,01)	1,33 ± (0,06)	1,68 ± (0,04)	1,83 ± (0,05)
UrHap20	0,45 ± (0,04)	1,12 ± (0,05)	0,84 ± (0,01)	1,36 ± (0,10)	0,80 ± (0,04)
TPSUr/Hap50	0,73 ± (0,07)	1,41 ± (0,05)	1,25 ± (0,03)	1,03 ± (0,09)	1,16 ± (0,06)
TPSUr/Hap20	0,60 ± (0,04)	1,11 ± (0,04)	1,61 ± (0,03)	1,65 ± (0,05)	1,63 ± (0,03)

^1^Mean values reported from three replicate soil cores, with standard deviations in parentheses

**Table 5:** Available P in soil fraction extracted with water (P-water) and extracted by anion resin (P-resin) after the aerobic incubation period SSP of Hap and nanocomposites compared to the P applied to the soil.

Treatment	% P available			
	P-water		P-resin	
	0 day	42 days	0 day	42 days
Hap	21.0 ab^1^	2.9 c	96.2 a	44.1 d
SSP	25.9 a	3.4 c	91.8 a	49.1 d
UrHap20	19.6 b	25.6 a	97.5 a	87.3 a
UrHap50	19.9 b	22.3 a	81.9 a	88.6 a
TPSUr/Hap20	11.4 c	0.7 d	76.0 a	62.6 bc
TPSUr/Hap50	4.6 d	10.6 b	69.2 a	73.8 ab

^1^Mean values reported from triplicate incubations. Values within a column followed by the same letter do not differ significantly by the Duncan’s test at a significance level of 0.05.

**Table 6:** pH value after aerobic incubation period of soil, SSP, Hap and nanocomposites in soil.

Treatment	pH after incubation period		∆ pH 2	
	0 day	42 days	0 day	42 days
Soil	5,19 ± (0,04)^1^	4,94 ± (0,03)	—	—
Hap	6,09 ± (0,04)	4,85 ± (0,02)	0,90	−0,08
SSP	4,91 ± (0,02)	5,24 ± (0,04)	−0,28	0,30
UrHap20	6,36 ± (0,03)	7,20 ± (0,01)	1,17	2,26
UrHap50	7,09 ± (0,05)	7,90 ± (0,05)	1,90	2,97
TPSUr/Hap20	5,84 ± (0,01)	6,30 ± (0,06)	0,65	1,37
TPSUr/Hap50	6,31 ± (0,02)	7,58 ± (0,06)	1,12	2,65

^1^Mean values reported from three replicate soil cores, with standard deviations in parentheses.

^2^Difference in pH of each treatment relative to soil.
